# A Ray of Hope in Darkness: A Case Report on the Rehabilitation of a Patient With Sudden Visual Impairment Due to Traumatic Optic Neuropathy

**DOI:** 10.7759/cureus.70572

**Published:** 2024-09-30

**Authors:** Gaurav Paramasivan, Gnanapoonkodi Bhaskaran, Sarika Gopalakrishnan, Durga Priyadarshini Santhakumar, Rajiv Raman

**Affiliations:** 1 Optometry, Sankara Nethralaya, Chennai, IND; 2 Shri Bhagwan Mahavir Vitreoretinal Services, Sankara Nethralaya, Chennai, IND; 3 Optometry, Low Vision Care Clinic, Sankara Nethralaya, Chennai, IND; 4 Neuro-Ophthalmology, Sankara Nethralaya, Chennai, IND

**Keywords:** low vision care clinic, low vision devices, rehabilitation, saarthi, s: traumatic optic neuropathy

## Abstract

Traumatic optic neuropathy (TON) refers to optic nerve damage resulting from trauma to the eye or head. Loss of visual functions is the hallmark of all optic neuropathies. Due to the sudden loss of vision, even after treatment, patients feel physically and psychologically disturbed. Hence, they are referred to low vision care practitioners for appropriate care and counselling services. The patient undergoes a series of examinations to assess their current condition and specific needs. Based on the findings, the patient was advised on low vision devices (LVDs) and rehabilitation services to help them utilize their residual vision. A 35-year-old man diagnosed with TON reported to a low vision clinic with visual acuity of no perception of light in the right eye and 6/60p, N12 @ 20cm in the left eye for assistive devices and further rehabilitation. A near vision trail was given with +10.00D half-eye spectacles, a 7x illuminated stand magnifier, and Niki closed-circuit television (CCTV). There was an improvement in N6 vision for near with Niki CCTV. Following this, the patient was referred to the rehabilitation training center, where he received counselling and tailored training based on his needs, including orientation and mobility, mobile and assistive technology usage, independent living skills, and job rehabilitation. These methods enhance the patient's ability to navigate daily life more independently, helping him regain confidence and functionality. With proper rehabilitation and LVDs, TON patients can lead an independent and confident life.

## Introduction

Traumatic optic neuropathy (TON) is a form of optic neuropathy in which the optic nerve is damaged by trauma to the eye or head [1]. The worldwide incidence of TON varies from 0.7 to 2.5%, with a higher incidence of 4.04% reported in India [2-4]. In a study conducted by Yadav et al., the incidence of TON was found to be 1.96% in the North Indian population [5]. According to a survey by Lee et al., the prevalence of TON in the general population of India is one in 1,000,000 (million) [6,7]. Loss of visual function is a hallmark of all optic neuropathies. The clinical findings of TON include vision loss, visual field defect, relative afferent pupil defect (RAPD) in asymmetric cases, and color vision defect [8]. Approximately 500,000 patients report unilateral traumatic blindness every year [9]. It is calculated that approximately 0.55% of closed head injuries, 2.5% of patients with mid-face fractures, and 10% of patients with craniofacial fractures develop TON [7,9,10]. The common causes of adult TON are motor vehicle and bicycle accidents (49%), followed by falls (27%) and assaults (13%) [11,12].

Optic nerve injuries due to trauma are classified into two categories: direct and indirect. In indirect injuries, concussive forces are transmitted to the optic nerve due to cranial or orbitofacial trauma. The most frequent location for optic nerve injury is intracanalicular, followed by the intracranial optic nerve. On the other hand, direct injuries involve external objects penetrating the tissues and damaging the optic nerve, though they are less common than indirect optic neuropathy [5].There is no treatment consensus available for TON, and the prognosis is guarded [13]. Treatments that have been attempted for TON include careful observation, systemic corticosteroid therapy, and surgical decompression of the optic nerve [11,13]. Patients are usually anxious, depressed, and detached from society. They are referred to low vision clinics (LVCs) and low vision care practitioners to guide patients to improve their residual vision with the help of appropriate low vision care and counselling services [14]. There are many low vision equipment and rehabilitation services available for patients to utilize their residual vision. This encourages patients to improve psychologically and lead a better quality of life.

Here, we report a case of TON managed with low vision aids and rehabilitation services and their impact on the patient’s life. To our knowledge, no literature on the options of low vision devices (LVDs) and rehabilitation is available and suitable for patients with TON. This case underscores the long-term impact of integrating rehabilitative care and also shifts the focus from merely addressing the condition to improving the patient's overall well-being, independence, and daily living skills, distinguishing this case from more traditional TON treatments.

## Case presentation

A 35-year-old man, presented with a history of diminished vision in both eyes for the past one year following trauma. He was previously diagnosed with TON in both eyes. He had been injured in a road traffic accident (RTA) while riding his bike without wearing a helmet and came for further management at a tertiary care eye center. He had a history of ear bleeding and loss of consciousness (LOC) for two months following RTA. His general health was good, although he experienced memory disturbances. A computed tomography scan of the brain showed contusions affecting both frontal lobes, temporal lobes and some facial bones (right sphenoid and zygomatic).

On ocular examination, the best-corrected visual acuity in the right eye was no perception of light (No PL), and in the left eye, it was 6/60, near vision N36 with effort at 15-20 cm using the Snellen and N notation charts. On retinoscopy, the glow was clear in both eyes. The acceptance in the right eye was +0.00 diopters sphere (DS) and the left eye was +0.00DS/-0.75 diopters cylinder (DC)X 90, N36 with effort. The external face examination was normal. The slit-lamp examination was unremarkable except for early cataract changes in both eyes. The right eye had an RAPD and the left eye had an ill-sustained pupil. The Hirschberg reflex showed right hypertropia, and the extraocular movements were full and free. The color vision using the Ishihara pseudoisochromatic plates was impaired in the left eye. Intraocular pressures were 14 mmHg and 15 mmHg in the right eye and left eye, respectively, by applanation tonometry. Indirect ophthalmoscopy showed optic atrophy in both eyes with an old vitreous hemorrhage in the left eye, and the rest of the retina and macula was normal. Humphrey Visual Field assessment of the left eye using FastPac 30-2 showed constricted fields.

Following the trauma, the patient experienced visual disturbances in both eyes, as mentioned earlier. Additionally, the presence of RAPD in the right eye signifies asymmetric optic nerve damage, with the right being more affected than the left. The patient had constriction of the visual fields in the left eye, which further reduces the patient's visual capacity, resulting in significant vision impairment. The patient was explained about the guarded visual prognosis and given the options of low vision aids in the left eye. He was referred to a LVC for further evaluation and management. The results are summarized in Table 1.

**Table 1:** Comprehensive clinical findings of the patient. TON: traumatic optic neuropathy; DS: diopters sphere; DC: diopters cylinder; BCVA: best-corrected visual acuity; PL: perception of light; RAPD: relative afferent pupillary defect.

Ocular findings	Right eye	Left eye
Diagnosis	TON	TON
Nature of retinoscopy reflex	Clear	Clear
Objective refraction	+0.00 DS	+0.00 DS/-0.75 DCX 90
Subjective refraction		
BCVA-distance	No PL	6/60
BCVA-near	-	N36 @ 15-20 cm
External face examination	Normal	Normal
Slit-lamp examination	Unremarkable except-early cataract changes	Unremarkable except-early cataract changes
Pupillary examination	RAPD	Ill-sustained pupil
Hirschberg reflex	Right hypertropia	
Extraocular movements	Full and free	Full and free
Color vision test-Ishihara chart	Deferred	Impaired
Intraocular pressure	14 mmHg	15 mmHg
Indirect ophthalmoscopy	Optic neuropathy	Optic neuropathy, old vitreous hemorrhage
Humphrey Visual Field assessment	Deferred	Constricted fields

**Figure 1:**
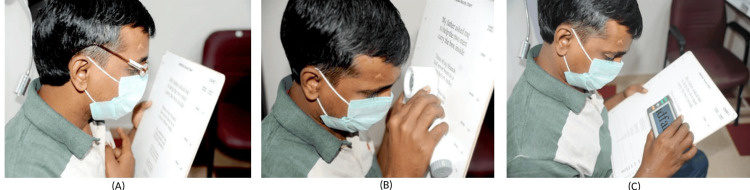
(A) LVD trails +10.00 D aspheric half eyes; (B) 7X illuminated stand magnifier; (C) Niki CCTV training. LVD: low vision device; CCTV: closed circuit-television.

He reported to the LVC after a month. The patient had worked as a software designer but he discontinued his job two years ago due to vision loss. His visual needs included improved computer use, distance vision, mobile use, and near vision. The left eye's presenting distance visual acuity was 6/60p on the Bailey-Lovie chart, and the near visual acuity in the left eye was N12p @ 20 cm using the MN read chart. A trial with +10.00 D aspheric half eyes (Figure 1A) revealed no improvement in near vision; however, a trial with a 7X illuminated stand magnifier (Figure 1B) demonstrated an improvement in near vision to N6 with effort. A trial with the Niki closed-circuit television (CCTV) (Figure 1C) was given, and his near vision improved to N6. He was advised to increase the task illumination for better contrast. A demonstration of the computer and mobile software was given. He was referred to a vision enhancement clinic for computer software and mobility training to motivate and improve his self-confidence.

**Figure 2:**
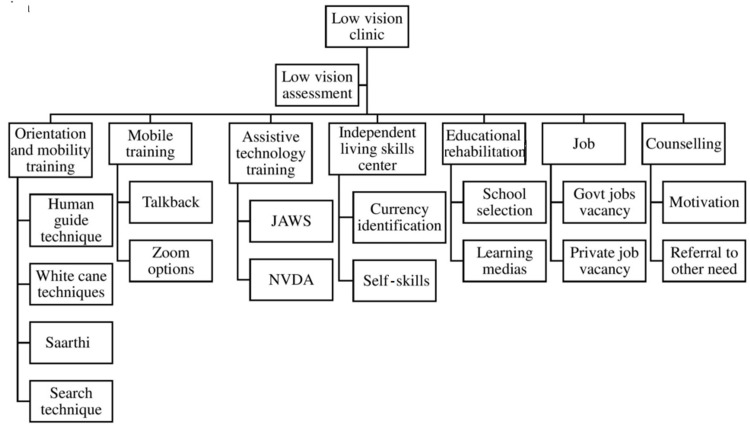
List of trainings given in the rehabilitative center for the visually impaired. NVDA: Non-Visual Desktop Access; JAWS: Job Access With Speech.

The training sessions were as follows: (1) orientation and mobility training; (2) mobile training; (3) assistive technology training, (4) independent living skills, (5) job rehabilitation, and (6) counselling (Figure 2). In addition to the training sessions, the patient and their companion were given guidance about environmental modifications and how to cope up with the color vision issues. 

**Figure 3:**
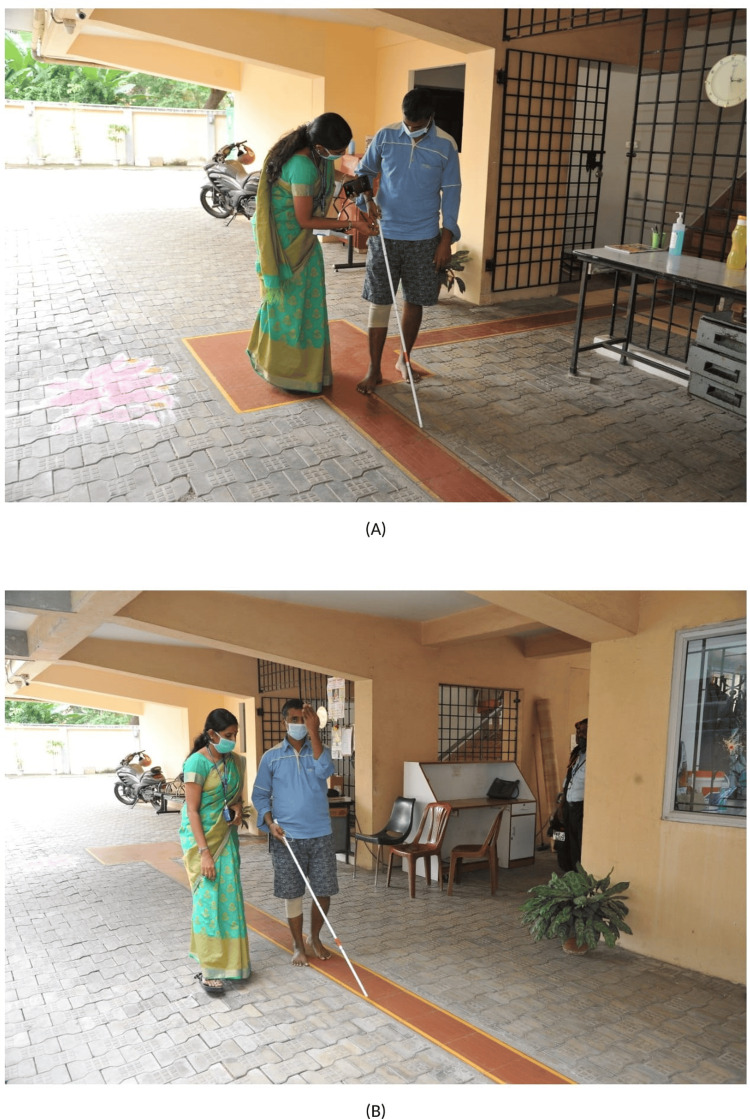
(A) Cane holding, arcing, touch and tap, three-point touch, and texture deduction training. (B) Search technique.

He was trained in various techniques, such as the human guide technique, white cane technique, Saarthi (assistive mobility device), search technique, and body protection techniques. Both outdoor and indoor mobility trainings were given. In the white cane technique, cane holding, arcing, touch and tap, three-point touch, and texture deduction training were given, as shown in Figure 3A,B. Saarthi assistive mobility device was trained. Saarthi is a mobility assistive device that detects and vibrates when there is an obstacle using its sensor [15]. It alerts visually impaired persons during movement. The patient became confident and also learned road crossing using the Saarthi device.

The patient was trained to use the built-in TalkBack feature on the Realme 2 Pro mobile phone. He received instruction on how to touch, listen, and identify the options. He was taught to access WhatsApp, YouTube, and dial numbers using TalkBack. The patient was trained with assistive software Job Access With Speech (JAWS), Non-Visual Desktop Access (NVDA), and MAGic on both desktop and laptop computers. Orientation on the keyboard and parts of the computer was given. He was trained in Microsoft Office with JAWS software as well as in using shortcut keys for Gmail and Google applications.

The patient was trained and motivated to use other senses for a better understanding of his surroundings. He was trained to do all day-to-day activities such as meal management, house management, financial management, family care management, personal care, and leisure time activities independently. Identifying and handling currency were also taught. The patient was educated regarding job openings in both government and private sectors and ideas regarding alternative businesses to sustain and support his family's income. Knowing and understanding the guarded visual prognosis, the patient and his family members were counselled and motivated. A series of counselling sessions were conducted to help him regain confidence in executing his daily activities. The patient has shown notable improvements, now walking and performing tasks independently. He also demonstrates confidence in using the computer and mobile with built-in modifications. He applied for a job with the help of Vision Enhancement Clinic and has an offer letter in hand.

## Discussion

Several studies have explored the preference for LVDs and their benefits in various ocular conditions such as Stargardt disease, Leber's hereditary optic neuropathy, and central or peripheral field loss [16,17]. However, to our knowledge, there is a lack of research specifically addressing the use of LVDs and rehabilitation services for patients with TON. Rehabilitation of patients with TON is a great challenge. Sudden loss of vision makes anyone disturbed both physically and psychologically. Dedicated rehabilitation for such patients motivates many of them to be independent. Our patient was one of those satisfied and motivated patients who volunteered to motivate many other such patients who visited LVC and rehabilitation centers. Although this was one case example, the subject’s positive feedback encouraged enrolling patients with TON for rehabilitation at an early stage. The quality of vision is less in patients with a constricted visual field. LVD (magnification) helps patients with poor vision use their residual vision [16]. Mobility becomes the most common problem with sudden visual loss. An adult with visual impairment needs to learn orientation and mobility skills to compensate for reduced visual information and to maintain travel independence [18]. One of the aims of vision rehabilitation is to maintain one's independence of travel by teaching visually impaired adults to ambulate and negotiate the environment safely and independently [18].

Previous studies on assistive technology indicate that computer assistive technology is considered an important tool to achieve educational, employment, and personal goals for the visually impaired [19]. The "Persons with Disabilities Act" gives prominence to equal educational opportunities for the blind through assistive technology [20]. The service providers play a major role, as the success or failure of the service is mainly influenced by them. They should be well trained since each visually impaired person is unique, and hence, different strategies are needed to be adapted to get a positive outcome.

LVDs are recommended based on the patient's visual state because all devices have certain advantages and disadvantages. A previous study on visual rehabilitation in Stargardt disease found that the most preferred LVD was a dome magnifier. Contradictorily, a study on LVD preference in patients with central field loss and peripheral field loss found that the spectacle magnifier was the most preferred magnifier [16]. Similarly, our patient also preferred a spectacle magnifier. When compared to other LVDs, the half-eye spectacle has a greater field of view and is more cosmetically acceptable. It can be utilized for writing tasks because it allows one to handle the material hands-free (pen, etc.). The disadvantages of a half-eye spectacle include a short working distance, limited lighting, and a limited depth of field. It is challenging for patients who have postural problems. The stand magnifier is small and lightweight. There is a high magnification option. It is appropriate for extended reading, and self-illumination is available. The downsides are a poor field of view and an unpleasant appearance. It is challenging for those who have posture issues or unsteady hands.

The development of electronic portable devices has led to a notable improvement in the visual acuity of patients with severe visual impairment [16]. Similarly, our patient's near vision improved with Nikki CCTV. The other LVD is Niki CCTV, and its advantages are maximum magnification, illumination level, operating distance, and text contrast. It can be utilized for a long duration of reading as well as spotting jobs. CCTV has minimal drawbacks, such as the need for training and adaptation for the comfort of use. Since the patient relied heavily on his family for mobility, he was trained to use the Saarthi device to enhance his independence. After completing the training, the patient began visiting the clinic on his own, without assistance from his family members.

## Conclusions

Rehabilitation for patients with TON presents a significant challenge due to the sudden and severe loss of vision, which affects both physical and psychological well-being. Nevertheless, dedicated rehabilitation services are crucial in restoring patients' independence and confidence. Evidence from case studies, such as one patient who, after a positive rehabilitation experience, actively engaged in motivating others, underscores the potential for early intervention to significantly improve outcomes for TON patients. This early support is essential, as vision loss often results in substantial mobility difficulties, necessitating the acquisition of orientation and mobility skills to maintain travel independence. Assistive technologies, including various LVDs, play a vital role in enhancing visual function and overall quality of life. Each device, from half-eye spectacles to stand magnifiers and CCTV systems, offers distinct advantages and limitations, thus emphasizing the importance of tailoring the selection to individual patient needs. A notable limitation is that advanced LVDs, such as virtual reality and augmented reality devices, were not tried. In future research, we can try the advanced LVDs and also check the quality of life after rehabilitation in a large sample. In conclusion, a comprehensive approach that incorporates personalized rehabilitation strategies, timely intervention, and effective use of assistive technologies is critical for improving the lives of visually impaired individuals, facilitating greater independence and enhanced quality of life.
